# Sclerostin Antibody Treatment Stimulates Bone Formation to Normalize Bone Mass in Male Down Syndrome Mice

**DOI:** 10.1002/jbm4.10025

**Published:** 2017-12-29

**Authors:** Diarra K Williams, Sean G Parham, Eric Schryver, Nisreen S Akel, R Shane Shelton, Jessica Webber, Francis L Swain, Jami Schmidt, Larry J Suva, Dana Gaddy

**Affiliations:** ^1^ Department of Veterinary Physiology and Pharmacology College of Veterinary Medicine and Biomedical Sciences Texas A&M University College Station TX USA; ^2^ Department of Orthopaedic Surgery Center for Orthopaedic Research University of Arkansas for Medical Sciences Little Rock AR USA; ^3^ Department of Veterinary Integrative Biosciences College of Veterinary Medicine and Biomedical Sciences Texas A&M University College Station TX USA

**Keywords:** DISEASES AND DISORDERS OF/RELATED TO BONE, PRECLINICAL STUDIES, ANABOLICS, WNT/β‐CATENIN/LRPs, BONE QCT/MCT

## Abstract

Down syndrome (DS), characterized by trisomy of human chromosome 21, is associated with a variety of endocrine disorders as well as profound skeletal abnormalities. The low bone mass phenotype in DS is defined by low bone turnover due to decreased osteoclast and osteoblast activity, decreasing the utility of antiresorptive agents in people with DS. Sclerostin antibody (SclAb) is a therapeutic candidate currently being evaluated as a bone anabolic agent. Scl, the product of the sclerostin gene (*SOST*), inhibits bone formation through its inhibition of Wnt signaling. SclAb increases bone mass by suppressing the action of the endogenous inhibitor of bone formation, Scl. To examine the effects of SclAb on the DS bone phenotype, 8‐week‐old male wild‐type (WT) andTs65Dn DS mice were treated with 4 weekly iv injections of 100 mg/kg SclAb. Dual‐energy X‐ray absorptiometry (DXA), microCT, and dynamic histomorphometry analyses revealed that SclAb had a significant anabolic effect on both age‐matched WT littermate controls and Ts65Dn DS mice that was osteoblast mediated, without significant changes in osteoclast parameters. SclAb treatment significantly increased both cortical and trabecular bone mass at multiple sites; SclAb treatment resulted in the normalization of Ts65Dn bone mineral density (BMD) to WT levels in the proximal tibia, distal femur, and whole body. Ex vivo bone marrow cultures demonstrated that SclAb increased the recruitment of the mesenchymal progenitors into the osteoblast lineage, as indicated by increased alkaline phosphatase–positive colonies, with no effect on osteoclast differentiation. Together, in the setting of a murine model of DS and decreased bone turnover, SclAb had a potent anabolic effect. SclAb stimulated bone formation and increased osteoblastogenesis without affecting osteoclastogenesis or bone resorption. These data suggest that SclAb is a promising new therapy to improve bone mass and reduce fracture risk in the face of the low bone mass and turnover prevalent in the DS population. © 2017 The Authors *JBMR Plus* published by Wiley Periodicals, Inc. on behalf of American Society for Bone and Mineral Research.

## Introduction

Down syndrome (DS), the trisomy of human chromosome (Hsa) 21, is the most common symptomatic chromosomal abnormality compatible with survival into adulthood.^1^ The likelihood of trisomy 21 is strongly associated with maternal age,^2^ although chromosomal copy of trisomy 21 can originate maternally or paternally. Indeed, individuals with DS present with a multifaceted disorder with more than 80 clinically defined phenotypes affecting virtually all organ systems.^3^ People with DS have some degree of cognitive impairment, various endocrine disorders and decreased fertility, as well as an abnormal pattern of skeletal growth in the long bones during adolescence.^4^ In addition, individuals with DS also exhibit a profound dysregulation of the adult appendicular skeleton, that we and others have reported.^5^, ^6^, ^7^, ^8^ It is clear that children and adults with DS exhibit a reduction in bone mineral density (BMD)^5^, ^6^, ^7^, ^8^, ^9^, ^10^ and an imbalance between bone resorption and formation during remodeling,^5^ contributing to the high incidence of osteopenia and osteoporosis in DS.^11^, ^12^

Unlike other low bone mass scenarios, the low BMD of DS is not associated with increased bone turnover but is instead the result of decreased bone turnover,^5^ despite the sustained and profound hypogonadism.^7^, ^13^ In this setting, antiresorptive treatments are not appropriate, making the need for anabolic agents that can enhance bone mass and strength particularly important. We have shown previously that intermittent parathyroid hormone (PTH) treatment of Ts65Dn DS mice induces a profound and significant elevation of bone mass and strength.^14^ PTH as an anabolic therapy has distinct limitations, including being limited to 24 months of treatment because of concerns regarding a potential link to osteosarcoma.^15^ It is important to recognize that no connection with the occurrence of osteosarcoma in humans currently exists between elevated serum PTH in hyperparathyroidism or PTH treatment.^16^ In the case of adults with DS, PTH is approved only for the treatment of osteoporosis in men and postmenopausal women who are at high risk for a fracture and not for use in younger adults. Therefore, there is a distinct opportunity and unmet need for the use of bone anabolic agents in DS.

Currently, the bone anabolic target receiving the most pharmaceutical attention is the inhibition of the sclerostin pathway. Sclerostin, the product of the *SOST* gene, produced mainly by osteocytes, is a potent negative regulator of bone formation via inhibition of the Wnt signaling pathway.^17^ Although a promising treatment candidate that increases bone mineral density and bone formation with decreased bone resorption in postmenopausal women with low bone mass,^18^ the potential utility of antisclerostin therapy to increase bone mass in challenging patient populations such as DS is unknown.

To model the potential utility of this therapeutic approach in DS, the effect of such an intervention was determined in this study. Sclerostin antibody treatment significantly stimulated bone mass in wild‐type mice and normalized bone mass in Ts65Dn DS mice, via a mechanism that was osteoblast‐mediated with little or no impact on osteoclastogenesis. The data suggest that bone anabolic therapies such as SclAb may be appropriate in healthy adult DS patients with low BMD.

## Materials and Methods

### Study design

Using baseline body mass to minimize intragroup differences, 8‐week‐old male Ts(17^16^)65Dn (Ts65Dn) mice and wild‐type (WT) mice (Jackson Laboratory, Bar Harbor, ME, USA) were randomly assigned to treatment (SclAb) or control vehicle‐treated (Vehicle) (*n* = 4–6/group each) for a total of four groups with all animals assigned and housed individually by genotype: group 1, WT (placebo); group 2, WT‐SclAb; group 3, Ts65 (placebo); group 4, Ts65‐SclAb. The groups received either vehicle (isotonic vehicle buffer) or SclAb (100 mg/kg/wk, both kindly provided by Dr Michaela Kniessel, Novartis, Basel, Switzerland) as weekly iv injections in the morning on days 0, 7, 14, and 21 as previously described^19^, ^20^ before euthanization on day 28. Measurements of body weight occurred at the same time per week to allow calculation of SclAb dose. All mice were maintained on a 12‐hour light/dark cycle, had *ad libitum* access to standard laboratory rodent chow and water, and were euthanized by CO_2_ inhalation at the end of the experiment (mice at 12 weeks of age). Only male mice were used in this study because of the subfertile nature of Ts65Dn male mice and the lack of commercial availability of Ts65Dn female mice due to the importance of female mice in colony maintenance. All animal procedures were approved by and performed in accordance with the guidelines of the University of Arkansas for Medical Sciences (UAMS) Institutional Animal Care and Use Committee (IACUC).

### Bone mineral density

Dual‐energy X‐ray absorptiometry (DXA, PIXImus II, GE Lunar Corp., Madison, WI, USA) was used to measure total body (excluding the head region), hindlimb, and spine BMD (g/cm^2^) as we have previously described.^14^, ^21^ Measurements were acquired at baseline and at the end of the study. Subregion analysis of the midshaft of the tibia of all mice was also performed.^21^ The precision of DXA in our laboratory is 1.7%.^22^, ^23^

### Analysis of trabecular and cortical bone by micro‐computed tomography (microCT)

Formalin‐fixed tibias were imaged using high‐resolution microcomputed tomography (μCT40, Scanco Medical, Brüttisellen, Switzerland). Briefly, the proximal tibia and tibial midshaft regions were scanned as 12‐μm isotropic voxel size using 55 kVp, 114 mA, and 200 ms. Bone volume fraction (BV/TV, %), trabecular thickness (Tb.Th, mm), trabecular separation (Tb.Sp, mm), trabecular number (Tb.N, 1/mm), connectivity density (ConnD 1/mm3), and structure model index (SMI) were calculated using previously published methods.^24^ The cancellous bone region was obtained using a semi‐automated contouring program that separated cancellous from cortical bone. At the midshaft of the tibia, total cross‐sectional area (CSA, mm^2^), medullary area (MA, mm^2^), and cortical thickness (Ct.Th, mm) were assessed in a 1‐mm‐long region centered at the midshaft. Bone was segmented from soft tissue using the same threshold for all groups, 245 mg HA/cm^3^ for trabecular and 682 mg HA/cm^3^ for cortical bone. All microCT scanning and analyses were compliant with published American Society for Bone and Mineral Research (ASBMR) guidelines for rodents.^25^

### Histology and bone histomorphometry

Quantitative static and dynamic histomorphometry was performed on paraffin and methyl methacrylate‐embedded tibias as we have previously described.^21^, ^22^, ^24^ Calcein (15 mg/kg) and alizarin red complexome (40 mg/kg) were injected intraperitoneally 7 and 2 days, respectively, before euthanization. Histomorphometric measurements were performed on the secondary spongiosa of the proximal tibia metaphysis using OsteoMeasure (Osteometrics, Atlanta, GA, USA). Static measurements in 4‐μm sections included osteoblast surface (Ob.S/BS, %) and osteoclast surface (Oc.S/BS, %) as we have previously described.^21^, ^24^ For dynamic histomorphometry, mineralizing surface per bone surface (MS/BS, %) and mineral apposition rate (MAR, μm/d) were measured in unstained sections under ultraviolet light and used to calculate bone formation rate with surface referent (BFR/BS, μm^3^/μm^2^/d). Terminology and units adhere to the recommendations of the histomorphometry nomenclature committee of the ASBMR.^26^

### Ex vivo bone marrow cultures

Bone marrow cells were harvested from femurs as previously described.^27^ In brief, for osteoclastogenesis, cells were flushed from femurs, washed, and cultured in 24‐well plates (Becton Dickinson Labware) at a density of 2 × 10^6^ cells per well in α‐minimal essential medium (α‐MEM), supplemented with 15% fetal calf serum and 10^−8^ M 1,25‐dihydroxyvitamin D3 (1,25(OH)2D3) in quadruplicate wells per treatment. Cells were fed every 3 days with half‐volumes of medium, until day 10, when cells were fixed and stained with tartrate‐resistant acid phosphatase (TRAP) to facilitate determination of the number of TRAP‐positive multinucleated (3 or more nuclei) cells formed per well. For osteoblastogenesis, bone marrow cells harvested from femurs were seeded in triplicate at 1 × 10^6^ cells/well in 12‐well tissue culture plates (Becton Dickinson Labware) containing osteoblast medium (α‐MEM +15% FBS containing 10 mM BGP and 50 μM ascorbic acid). The recruitment of mesenchymal progenitors into the osteoblastic lineage was determined by alkaline phosphatase‐positive staining for colony‐forming unit‐fibroblast (CFU‐F) on day 10 and the total number of colonies after staining with methyl green. Both the number of AP(+) colonies and total colonies were enumerated as described.^27^

### Statistical analysis

Based on an expected stimulation of bone volume in the SclAb‐treated groups of greater than 12%^28^, ^29^ a power analysis before the start of this study suggested *n* = 5 per group would provide sufficient power (0.8) to detect a significant effect of SclAb treatment. All data points were checked for normality and standard descriptive statistics computed. Comparisons within genotype (WT or Ts65Dn) were performed using Student's *t* test as appropriate. Pairwise comparisons of treatment effects were evaluated using analysis of variance (ANOVA) followed by Tukey's method to test for differences between all groups using Prism 5 software (GraphPad Software, San Diego, CA, USA). Data are presented as mean ± SD and differences were considered significant at *p* < 0.05 and are reported as such. Box and whiskers plots display the median, quartiles, and extremes of the data with individual data points displayed to show the distribution of the data.

## Results

### Normalization of BMD by sclerostin antibody treatment at multiple sites in Ts65Dn mice

**Figure 1:**
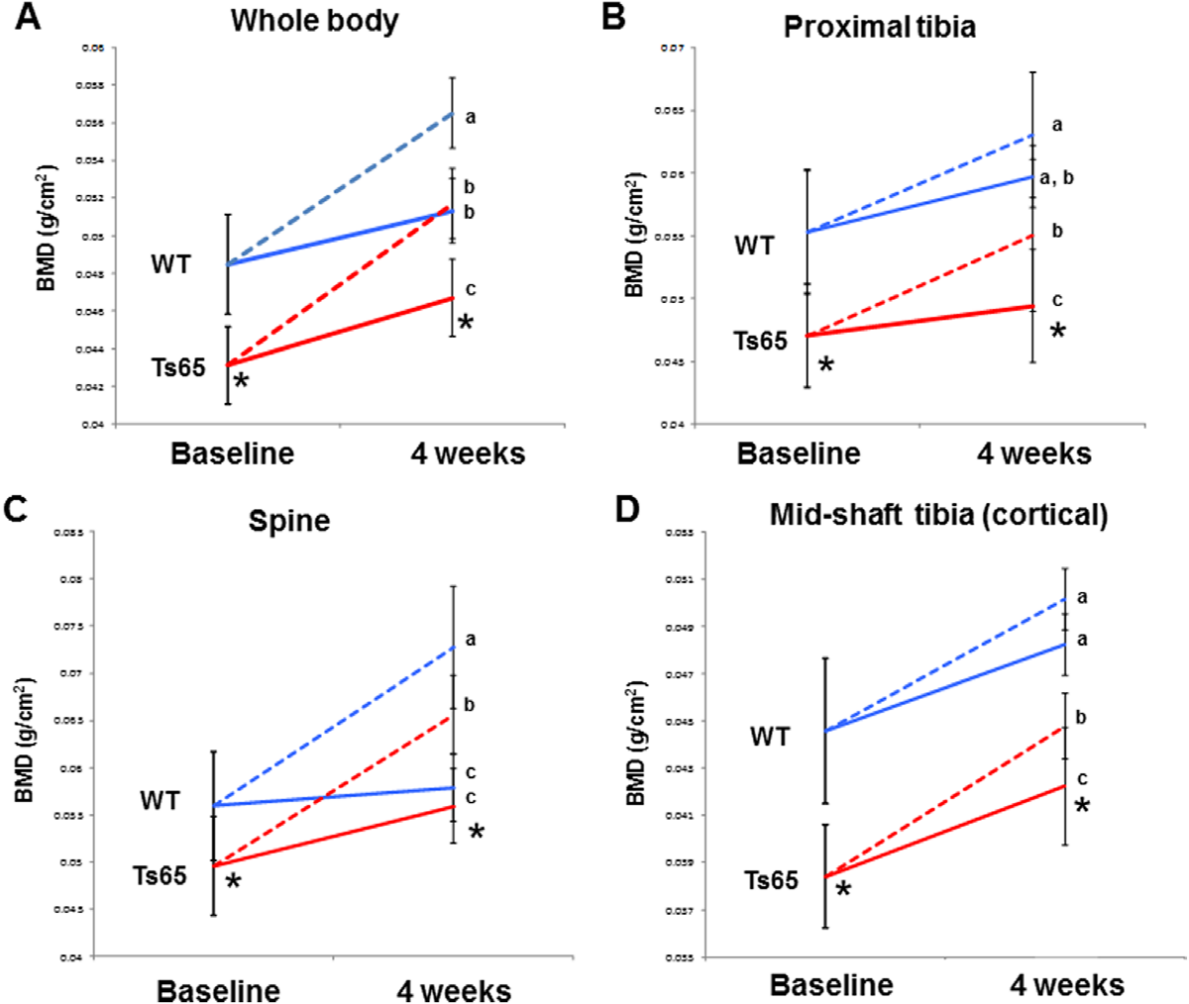
Sclerostin antibody normalizes low BMD in Ts65Dn DS mice. BMD was measured via DXA at baseline and after treatment (week 4) at the whole body (*A*), proximal tibia (*B*), spine (*C*), and tibia midshaft (cortical) (*D*) sites. Vehicle treated = solid line; SclAb treated = dashed line. *Indicates Ts65Dn vehicle treated significantly different from WT vehicle treated at both baseline and at 4 weeks (*p* < 0.05). Different letters are significantly different from each other at *p* < 0.05.

At baseline, Ts65Dn mice body weight was significantly less than WT (*p* < 0.001) and the genotype‐specific differences in body weight remained apparent at the end of the experiment (4 weeks) (*p* = 0.01)^14^ (data not shown). As shown previously,^14^ BMD of vehicle‐treated Ts65Dn mice was significantly reduced from WT vehicle‐treated mice at baseline and at 4 weeks (*p* < 0.001 versus WT at baseline; *p* < 0.05 at 4 weeks). After weekly SclAb treatment (100 mg/kg/wk) whole body, spine, tibia (Fig. 1), and femur (data not shown) BMD significantly increased in both WT (*p* < 0.001 versus vehicle‐treated WT) and Ts65 mice (*p* < 0.001 versus vehicle‐treated Ts65Dn) after 4 weeks. Remarkably, at both the whole body and proximal tibia, the low bone mass phenotype of Ts65Dn mice normalized after 4 weeks of SclAb treatment and was not different from vehicle‐treated WT mice (Fig. 1 *A*, *B*). In contrast, Ts65Dn spine BMD was significantly increased compared with WT controls 4 weeks after SclAb treatment (Fig. 1 *C*). At the primarily cortical tibia midshaft, SclAb treatment significantly increased cortical BMD in both genotypes, but Ts65Dn cortical BMD was normalized (Fig. 1 *D*). The extent and rate of increase in BMD at all sites in SclAb‐treated animals was similar in both genotypes.

### Effect of sclerostin antibody on bone microarchitecture and geometry

**Figure 2:**
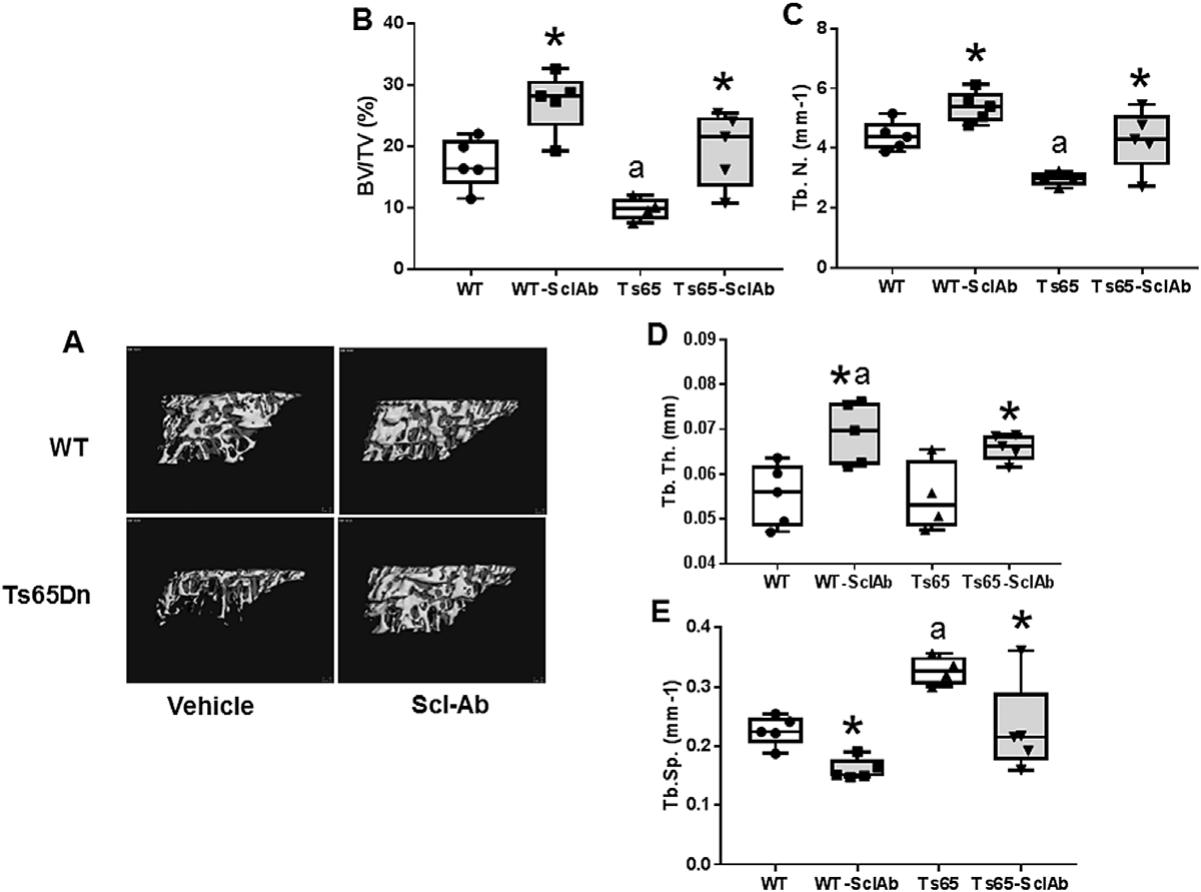
Sclerostin antibody treatment significantly improved bone microarchitecture assessed by microCT. (*A*) MicroCT renderings of the proximal tibia. (*B*) Measurement of BV/TV (%). *Shows a significantly higher BV/TV in SclAb‐treated mice compared with vehicle‐treated genotype controls (WT‐SclAb versus WT, *p* = 0.0169; Ts65‐SclAb versus Ts65, *p* = 0.028). ^a^Shows significantly different BV/TV compared with vehicle‐treated WT (WT versus Ts65, *p* = 0.0187). No difference in BV/TV is observed between WT and Ts65‐SclAb (*p* = 0.8415). (*C*) Measurement of Tb.N. (mm^−1^). *Shows a significantly higher Tb.N in SclAb‐treated mice compared with vehicle‐treated genotype controls (WT‐SclAb versus WT, *p* = 0.0159; Ts65‐SclAb versus Ts65, *p* = 0.041). ^a^Shows significantly different Tb.N. compared with vehicle‐treated WT (WT versus Ts65, *p* = 0.0227). No difference in Tb.N. is observed between WT and Ts65 SclAb (*p* = 0.9887). (*D*) Measurement of Tb.Th (mm). *Shows a significantly increased Tb.Th in SclAb‐treated mice compared with vehicle‐treated genotype controls (WT‐SclAb versus WT, *p* = 0.0167; Ts65‐SclAb versus Ts65, *p* = 0.020). No difference in Tb.Th is observed between WT and Ts65Dn at the genotype level (*p* = 0.9996). (*E*) Measurement of Tb.Sp (mm). *Shows a significantly decreased Tb.Sp in SclAb‐treated mice compared with vehicle‐treated genotype controls (WT‐SclAb versus WT, *p* = 0.0015; Ts65‐SclAb versus Ts65, *p* = 0.022). ^a^Shows significantly different Tb.Th compared with vehicle‐treated WT (*p* = 0.0178). No difference in Tb.Sp is observed between WT and Ts65 SclAb (*p* = 0.9994).

Overall, SclAb treatment improved trabecular bone microarchitecture and cortical bone geometry in both WT and Ts65Dn mice (Fig. 2 and Table 1). Trabecular microarchitecture was markedly enhanced in both WT and Ts65Dn mice treated with SclAb compared with VEH‐treated mice. MicroCT renderings (Fig. 2 *A*) as well as quantitation showed significant SclAb treatment differences within genotype of BV/TV (*p* = 0.025 WT; *p* = 0.008 Ts65; Fig. 2 *B*), Tb.N (*p* = 0.019 WT; *p* = 0.02 Ts65; Fig. 2 *C*), Tb.Th (*p* = 0.035 WT; *p* = 0.02 Ts65; Fig. 2 *D*), and Tb.Sp (*p *= 0.004 WT; *p* = 0.022 Ts65; Fig. 2 *E*). In cortical bone, the SclAb‐mediated effects were anabolic but not as consistently robust (Table 1). After SclAb treatment, cortical thickness was significantly increased in both genotypes and in Ts65Dn‐treated animals was restored to near WT levels. Medullary area was significantly lower in Ts65Dn animals compared with WT (*p* = 0.045) as we have previously shown.^14^ This parameter was significantly reduced in both WT and Ts65Dn mice by SclAb treatment (Table 1). In WT animals, treatment with SclAb also led to significantly increased total cross‐sectional area (Tt.CSA). In sum, these cortical changes were less profound than the changes observed in trabecular bone but appear to be due to endosteal bone apposition, as mice treated with SclAb had significantly increased cortical thickness (Ct.Th.) and decreased midshaft medullary area (MA) and diameter (MD) (Table 1).

**Table 1:** Effect of Sclerostin Antibody Treatment on Cortical Bone Geometry of the Tibia Assessed by MicroCT (Mean ± SD)

Parameter	Vehicle	SclAb	Vehicle	SclAb	*p* Value^a^	*p* Value^b^	*p* Value^c^
Ct.Th (mm)	0.270 ± 0.002	0.293 ± 0.006^a^	0.205 ± 0.003^c^	0.265 ± 0.005^b^	0.035	0.0002	<0.0001
MA (mm^2^)	0.122 ± 0.01	0.095 ± 0.005^a^	0.101 ± 0.005^c^	0.079 ± 0.01^b^	0.002	0.01	0.045
Endos. Pm (mm)	0.674 ± 0.089	0.728 ± 0.09	0.777 ± 0.06	0.706 ± 0.04	0.8	0.6	0.9
MD (mm)	0.355 ± 0.019	0.298 ± 0.03^a^	0.277 ± 0.015^c^	0.231 ± 0.03^b^	0.015	0.016	0.016
Tt.CSA (mm^2^)	0.419 ± 0.02	0.484 ± 0.05^a^	0.316 ± 0.008^c^	0.366 ± 0.03	0.0379	0.16	0.0018
Ps.Pm (mm)	1.576 ± 0.11	1.394 ± 0.169	1.293 ± 0.055	1.284 ± 0.09	0.4	0.9	0.088
AvD. Midshaft (mm)	0.600 ± 0.025	0.588 ± 0.03	0.545 ± 0.05	0.534 ± 0.02	0.9	0.9	0.1

Ct.Th =cortical thickness; MA = medullary area; Endos. Pm = endosteal perimeter; MD = medullary diameter; Tt.CSA = total cross‐sectional area; Ps.Pm = periosteal perimeter; AvD. midshaft = average diameter of midshaft.

^a^ *p* < 0.05 WT‐VEH versus WT‐SclAb.

^b^ *p* < 0.05 Ts65Dn vehicle control versus Ts65Dn SclAb.

^c^ *p* < 0.05 WT vehicle control versus Ts65Dn vehicle.

### Sclerostin antibody treatment increases osteoblast parameters without altering osteoclast parameters

**Figure 3:**
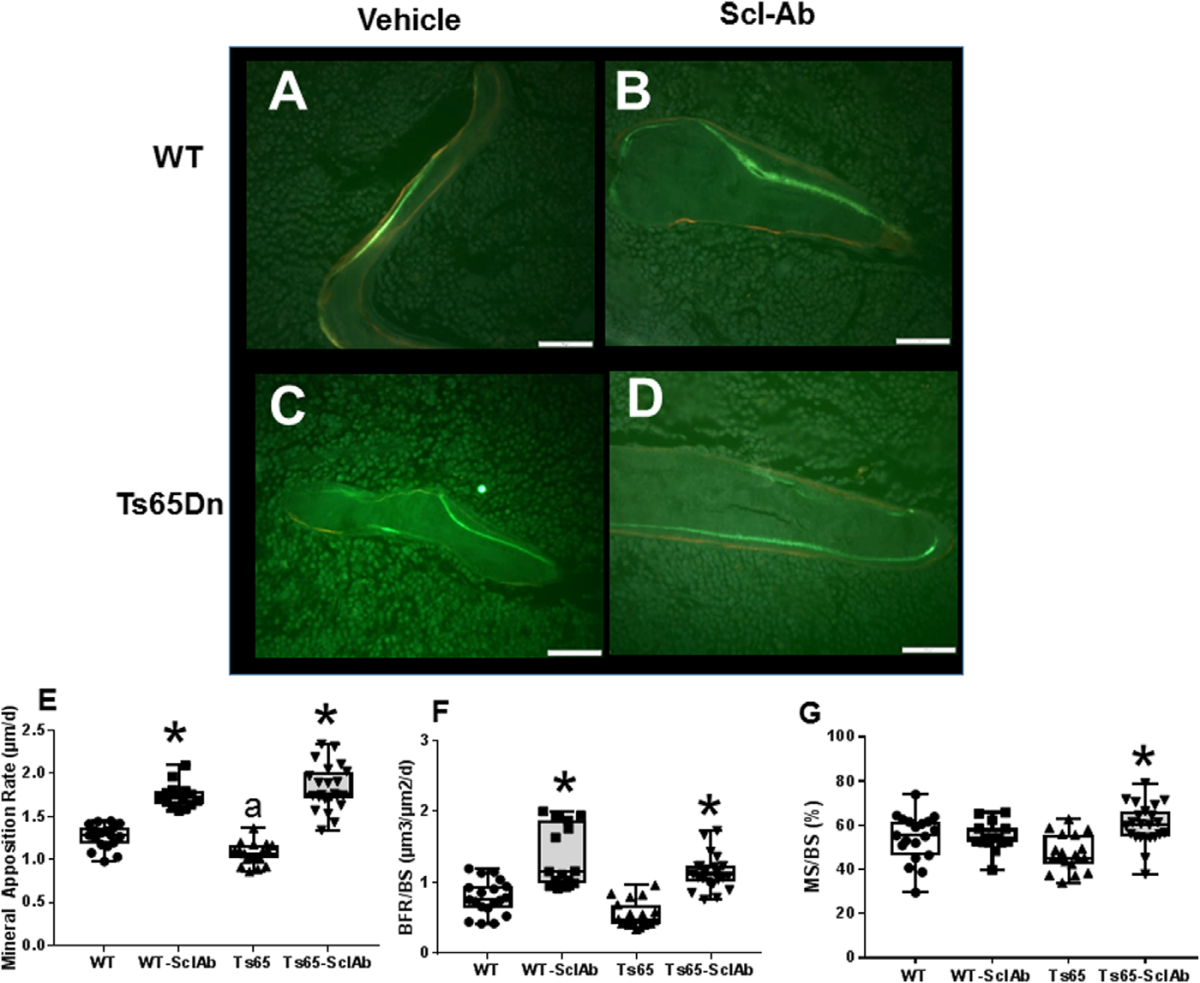
Sclerostin antibody treatment stimulates bone formation. Representative images of dual fluorochrome labeling of trabecular bone surfaces in (*A*) WT control (vehicle); (*B*) WT‐SclAb; (*C*) Ts65Dn (vehicle); (*D*) Ts65Dn‐SclAb. The increased distance between labels in mice treated with SclAb indicates bone anabolism. Images were acquired under fluorescent light using a 40× objective. Scale bar = 50 μm. Effect of vehicle (open bars) and SclAb treatment (gray bars) treatment on (*E*) mineral apposition rate (MAR, µm/d), (*F*) bone formation rate/bone surface (BFR/BS, µm^3^/µm^2^/d), and (*G*) mineral surface/bone surface (MS/BS, %) in WT and Ts65Dn DS mice. *p < 0.05 shows significant differences in SclAb‐treated mice compared with vehicle‐treated genotype controls. ^a^Shows significant difference in vehicle‐treated Ts65 compared with vehicle‐treated WT, *p* < 0.05. (MAR WT‐SclAb versus WT, *p* = 0.0093; Ts65 versus Ts65‐SclAb, *p* < 0.0001), (BFR/BS WT‐SclAb BFR/BS versus WT, *p* < 0.0001; Ts65 versus Ts65‐SclAb, *p* = 0.0007), (MS/BS WT‐SclAb versus WT, *p* = 0.9; Ts65 versus Ts65‐SclAb, *p* < 0.0001).

Histologic evaluation of nondecalcified double fluorochrome‐labeled murine tibias (Fig. 3 *A–D*) identified low bone formation in Ts65Dn compared with WT mice that was increased with SclAb. Quantitation of bone formation measured as mineral apposition rate (MAR) confirmed that MAR was significantly decreased in Ts65Dn mice compared with WT mice at baseline (Fig. 3 *E*) (*p* = 0.042).^14^ After treatment with SclAb, bone formation parameters BFR/BS and MAR were significantly elevated in both genotypes (Fig. 3 *E*, *F*). Interestingly, SclAb‐treated Ts65Dn animals had a significantly increased mineralizing surface to bone surface (MS/BS) (*p* < 0.0001) that was not found in SclAb‐treated WT animals, perhaps because of the SclAb‐related stimulation of the low osteoblast activity inherent in Ts65Dn mice and not regulated in WT animals.

SclAb treatment significantly increased osteoblast surface/bone surface (Ob.S/BS) in WT (19 + 4% versus SclAb 34 ± 5%; *p* = 0.0014) and in Ts65Dn mice (14 ± 4% versus SclAb 24 ± 3%; *p* = 0.045). However, SclAb treatment had no significant effect on osteoclast surface (Oc.S/BS) in either WT (0.9 ± 1% versus SclAb 1.1 ± 0.8%; *p* = 0.8) or Ts65Dn mice (2 ± 2% versus SclAb 3 ± 2%; *p *= 0.9), suggesting the profound anabolism of SclAb observed in both genotypes was independent of any detectable changes in osteoclast parameters in vivo.

**Figure 4:**
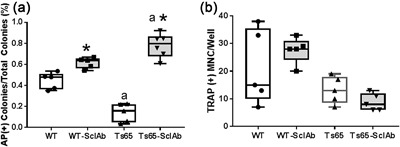
Sclerostin antibody increases osteoblast recruitment but not osteoclastogenesis. Ex vivo bone marrow cultures of bone marrow from vehicle‐ and SclAb‐treated WT and Ts65Dn mice. (*A*) Alkaline phosphatase positive (AP+)/total colonies are significantly less in Ts65Dn than WT at baseline (*p* = 0.0002). (AP+)/total colonies were significantly increased in both genotypes by SclAb treatment, indicative of increased recruitment into osteogenic lineage and differentiation of precursors by SclAb treatment. *Significantly different from genotype control (WT‐SclAb versus WT, *p* = 0.039; Ts65‐SclAb versus Ts65, *p* < 0.0001). ^a^Shows significantly different AP+/total compared with vehicle‐treated WT at *p* < 0.05 (Ts65‐SclAb versus WT, *p* < 0.0001). (*B*) Whole bone marrow cultured toward osteoclasts and TRAP+MNC/well enumerated. SclAb treatment has no significant effect on osteoclastogenesis.

Next, using ex vivo bone marrow cultures, the same osteoblast‐specific effects of SclAb treatment were observed. As in prior reports,^14^ recruitment of bone marrow cells into the osteoblast lineage measured as alkaline phosphatase positive (AP+) colonies/total colonies or the osteoclast lineage as TRAP‐positive multinucleated cells (Figure 4) were less in Ts65Dn at baseline. However, SclAb treatment significantly increased AP+ colonies/total colonies in both genotypes (Fig. 4 *A*) with no demonstrable effect on osteoclastogenesis (Fig. 4 *B*). Interestingly, the effect of SclAb on TS65Dn on AP+ colonies/total colonies was able to stimulate recruitment beyond WT or even WT treated with SclAb, perhaps indicative of the extent to which osteoblast lineage is suppressed in Ts65Dn and that can be activated by treatment.

## Discussion

In general, social and consenting issues and the apparent reluctance of pharmaceutical companies to treat the underserved DS population complicate pharmacologic intervention in at‐risk populations, such as people with DS. This is particularly relevant in the context of low bone mass, where people with DS are at increased risk of fracture.^30^ At present, there are no medical treatments for the low bone mass in DS approved by the US Food and Drug Administration. Therefore, low BMD in DS is currently managed by a combination of calcium and vitamin D supplements, physiotherapy, nutritional effects, exercise, and off‐label (and contraindicated) oral bisphosphonate use.^1^ Thus, there is an urgent need for alternative treatments to increase bone mass and strength in this vulnerable population.

In this study, treatment with SclAb not only stimulated bone mass in WT and Ts65Dn DS mice but also returned bone mass and bone microarchitecture to normal age‐matched WT levels at multiple skeletal sites. The data suggest that the anabolic effect of SclAb treatment that is well documented in high bone turnover scenarios such as osteoporosis^18^ and osteogenesis imperfecta^31^, ^32^ was able to enhance bone mass, bone quality, and bone formation in the face of low bone turnover. The microarchitectural changes in the trabecular and cortical compartments are entirely consistent with increased bone strength after SclAb treatment, although this was not specifically assessed. Furthermore, dynamic histomorphometry from the trabecular bone compartment demonstrated that the increased bone volume was attributable to increased bone formation indices with no apparent impact on osteoclastic parameters in either genotype. This phenotype was also captured in ex vivo bone marrow cultures from vehicle‐ and SclAb‐treated animals that showed increased recruitment into the osteogenic lineage with no effects on osteoclastogenesis.

The significant improvements in bone mineral density, bone microarchitecture, and geometry, as well as increased bone formation rate in SclAb‐treated animals are reminiscent of those previously reported by Fowler and colleagues^14^ in WT and Ts65Dn mice treated with PTH. However, unlike PTH, there is little data to suggest that SclAb therapy would be inappropriate for the treatment of low bone mass in people with DS. Indeed, there is data from a recent randomized phase 2a clinical trial in adults with moderate osteogenesis imperfecta^31^ that demonstrated safe and powerful anabolic effects in a younger adult population than previous SclAb clinical trials. In contrast, there is conflicting data in a preclinical murine model of severe osteogenesis imperfecta suggesting that SclAb treatment may be less effective in more severely affected osteogenesis imperfecta mice.^33^ In any case, the bone modeling–based concept of antisclerostin treatment may provide a promising approach to cover longer treatment periods as part of a long‐term medication strategy important for younger patients that enables phases of bone regeneration and formation without changes in bone resorption that could be of significant benefit in the setting of low bone turnover.

It should also be noted that a recent double‐blind phase 3 clinical trial of SclAb (romosozumab) versus the bisphosphonate alendronate, involving postmenopausal women with osteoporosis and a previous fracture, adjudicated serious cardiovascular adverse events in the romosozumab‐treated group compared with the alendronate group.^34^ In particular, it was noted that cardiac ischemic events and cerebrovascular events were likely driving this imbalance, although further evaluation is needed to determine the cause.^34^ This may be an important finding in the context of DS, with a minority of DS individuals having congenital heart malformations.^1^ In the event of the clinical use of SclAb in DS, close attention to potential cardiac events would certainly need to be addressed, as would further investigation of the function of sclerostin in the vasculature.

As with most preclinical studies, this study has several limitations, including the use of a single dosing regimen of SclAb, assessment of a single 4‐week time point, no active comparator, and although sufficiently powered, only 4 to 6 animals per group. Thus, we may have missed early increases in bone resorption parameters associated with SclAb treatment and not found changes in osteoclast number or activity that occur with age in Ts65Dn mice.^14^ Future studies of longer duration with additional time points, increased animal numbers, and active comparators that assess the extent to which bone mass is maintained in the low bone turnover setting would be highly informative and valuable. Such studies will provide important insight into the mechanisms that are responsible for the skeletal responses to SclAb in DS. Indeed, based on the sustained low bone turnover in DS, we posit that the low bone turnover will facilitate the extended maintenance of bone mass gained during SclAb treatment. However, despite these limitations, the current study provides the first evidence that SclAb is an effective treatment option for people with DS and low bone mass and potentially in other low bone turnover scenarios. Further investigation to determine the efficacy of SclAb in people with DS and low bone turnover will be required for eventual clinical application.

## Disclosures

All authors state that they have no conflicts of interest.
